# More than three times as many Indigenous Australian clients at risk from drinking could be supported if clinicians used AUDIT-C instead of unstructured assessments

**DOI:** 10.1186/s13722-022-00306-5

**Published:** 2022-04-05

**Authors:** James H. Conigrave, K. S. Kylie Lee, Paul S. Haber, Julia Vnuk, Michael F. Doyle, Katherine M. Conigrave

**Affiliations:** 1 NHMRC Centre of Research Excellence in Indigenous Health and Alcohol, Faculty of Medicine and Health, Central Clinical School, Discipline of Addiction Medicine, Level 6, King George V Building, Drug Health Services, 83-117 Missenden Road, Camperdown, NSW 2050 Australia; 2 The Edith Collins Centre (Translational Research in Alcohol, Drugs and Toxicology), Sydney Local Health District, Sydney, NSW Australia; 3 National Drug Research Institute, Faculty of Health Sciences, Curtin University, Perth, WA Australia; 4 La Trobe University, Centre for Alcohol Policy Research, Bundoora, Victoria Australia; 5 Burnet Institute, Melbourne, Victoria Australia; 6 Royal Prince Alfred Hospital, Drug Health Services, Sydney, NSW Australia; 7 Aboriginal Health Council of South Australia, Adelaide, SA Australia; 8 Adelaide Rural Clinical School, The University of Adelaide, Adelaide, SA Australia

**Keywords:** Unstructured drinking risk, Aboriginal and Torres Strait Islander, Alcohol consumption, AUDIT-C, Australia, Screening

## Abstract

**Background:**

Aboriginal and Torres Strait Islander (‘Indigenous’) Australians experience a greater burden of disease from alcohol consumption than non-Indigenous peoples. Brief interventions can help people reduce their consumption, but people drinking at risky levels must first be detected. Valid screening tools (e.g., AUDIT-C) can help clinicians identify at-risk individuals, but clinicians also make unstructured assessments. We aimed to determine how frequently clinicians make unstructured risk assessments and use AUDIT-C with Indigenous Australian clients. We also aimed to determine the accuracy of unstructured drinking risk assessments relative to AUDIT-C screening. Finally, we aimed to explore whether client demographics influence unstructured drinking risk assessments.

**Methods:**

We performed cross-sectional analysis of a large clinical dataset provided by 22 Aboriginal Community Controlled Health Services in Australia. We examined instances where clients were screened with unstructured assessments and with AUDIT-C within the same two-monthly period. This aggregated data included 9884 observations. We compared the accuracy of unstructured risk assessments against AUDIT-C using multi-level sensitivity and specificity analysis. We used multi-level logistic regression to identify demographic factors that predict risk status in unstructured assessments while controlling for AUDIT-C score.

**Results:**

The primary variables were AUDIT-C score and unstructured drinking risk assessment; demographic covariates were client age and gender, and service remoteness. Clinicians made unstructured drinking risk assessments more frequently than they used AUDIT-C (17.11% and 10.85% of clinical sessions respectively). Where both measures were recorded within the same two-month period, AUDIT-C classified more clients as at risk from alcohol consumption than unstructured assessments. When using unstructured assessments, clinicians only identified approximately one third of clients drinking at risky levels based on their AUDIT-C score (sensitivity = 33.59% [95% CI 22.03, 47.52], specificity = 99.35% [95% CI 98.74, 99.67]). Controlling for AUDIT-C results and demographics (gender and service remoteness), clinicians using unstructured drinking risk assessments were more likely to classify older clients as being at risk from alcohol consumption than younger clients.

**Conclusions:**

Evidence-based screening tools like AUDIT-C can help clinicians ensure that Indigenous Australian clients (and their families and communities) who are at risk from alcohol consumption are better detected and supported.

**Supplementary Information:**

The online version contains supplementary material available at 10.1186/s13722-022-00306-5.

## Introduction

While Aboriginal and Torres Strait Islander peoples (hereafter ‘Indigenous Australians’) are more likely to abstain from drinking than their non-Indigenous counterparts [1], they experience a greater burden of disease from alcohol consumption [2]. A long history of socio-economic disadvantage and discrimination stemming from British colonisation and then from discriminatory policies from Australian governments have made addictions and health problems more likely for Indigenous Australians [3, 4].

Indigenous Australians die from alcohol-related causes at more than five (5.5) times the rate of non-Indigenous Australians [2]. Alcohol consumption also places strain on Indigenous Australian communities through contributions to chronic diseases and disabilities (e.g., fetal alcohol spectrum disorders, and disabilities caused through injury) [5–7] and interpersonal violence [8, 9]. Brief intervention and/or treatment can help people reduce their consumption [10], but at-risk individuals must first be detected [11]. Structured screening tools such as the Alcohol Use Disorders Identification Test consumption questions (AUDIT-C) [12] can help clinicians systematically determine which clients are at risk from drinking alcohol and may require support [13]. But, in practice, many clinicians do not use formal screening tools when making drinking risk assessments.

Aboriginal Community Controlled Health Services are required to report the proportion of Indigenous Australian clients that are assessed for drinking risk (using any means) to the Australian government [13]. We refer to these assessments as ‘unstructured drinking risk’ assessments as clinicians can use any methodology to establish risk. While a clinician could choose to perform a formal screening assessment, they could also simply ask clients if they believe themselves to be at risk, or they could rely on subjective impressions [13]. Unstructured drinking risk assessments made in primary health settings have been found to be inaccurate for general populations [14–16]. But the degree of inaccuracy has never been quantified for Indigenous Australian clients. While many clinicians are systematic in their assessments, at the aggregate level it is not clear how unstructured drinking risk assessments are made, and what factors might bias these assessments. Clinicians might identify at-risk individuals in part based on whether clients violate perceived norms (e.g., if they drink more than peers). Thus, client demographics such as age and gender could be factors which bias unstructured drinking risk assessments.

In this paper we explore the accuracy of unstructured drinking risk assessments relative to AUDIT-C screening. We aimed to determine how frequently clinicians make unstructured risk assessments and use AUDIT-C with Indigenous Australian clients. We also aimed to determine the accuracy of unstructured drinking risk assessments relative to AUDIT-C screening. Finally, we aimed to explore whether client demographics (age, gender, and remoteness) influence unstructured drinking risk assessments. Using a large clinical dataset provided by 22 Aboriginal Community Controlled Health Services [17], we extracted all clinical sessions where the results of unstructured drinking risk assessments and client AUDIT-C score were both reported in the same two-month reference period. We examine the sensitivity and specificity of unstructured drinking risk assessments using AUDIT-C as the reference test. Using multi-level logistic regression, we explore whether demographic factors can explain why clinicians making unstructured assessments determine some users to be at risk but not others.

## Methods

This study was performed as part of a broader project to test whether training and support can increase AUDIT-C screening rates at Aboriginal Community Controlled Health Services [18]. The results of the primary outcomes from that project have been published elsewhere [17]. As this work is exploratory no hypotheses or methods specific to the research question have been pre-registered.

### Ethical approval

Approval was obtained from eight ethics committees across Australia: the Aboriginal Health and Medical Research Council of NSW Ethics Committee (NSW; project 1217/16), Central Australian Human Research Ethics Committee (project CA-17-2842), Human Research Ethics Committee of Northern Territory Department of Health and Menzies School of Health Research (project 2017-2737), Central Queensland Hospital and Health Service Human Research Ethics Committee (project 17/QCQ/9), Far North Queensland Human Research Ethics Committee (project 17/QCH/45-1143), The Aboriginal Health Research Ethics Committee, South Australia (SA; project 04-16-694), St Vincent’s Hospital Melbourne Human Research Ethics Committee (project LRR 036/17) and Western Australian Aboriginal Health Ethics Committee (WA; project 779).

### Dataset

Data were routinely collected from 22 Aboriginal Community Controlled Health services’ clinical practice management software (‘Communicare’) [17, 18]. Services were recruited who served at least 1000 unique Indigenous Australian clients per year. The full details of service recruitment have been published elsewhere [18]. The dataset included every clinical session for Indigenous Australian clients. To prevent potential confounding, we only used data from the baseline period of the broader study—prior to the commencement of a training and support program (aimed at increasing AUDIT-C screening rates) which was the primary focus of the broader trial [17, 18]. As wait-list control services had a longer baseline period more data were retained from that arm of the trial. Data were extracted for the period starting on the 29th of August 2016 and ending on the 13th of August 2019.

### Instruments

Demographics included client age (continuous) and gender, and service remoteness (ordinal; based on service location and Australian Bureau of Statistics classifications) [19]. We used the following strata for classifying remoteness: ‘urban’ (major cities and inner regional), ‘regional’ (outer regional and remote), and ‘remote’ (very remote) [19].

#### Unstructured drinking risk

Clinicians indicated client drinking risk status using one of the following categories: ‘Ex-drinker’, ‘Non-drinker’, ‘Within safe drinking limits’, or ‘Unsafe—needs intervention’. This variable is used as a key performance indicator for Aboriginal Community Controlled Health Services [13]. The Australian government does not restrict how clinicians make these assessments [13].

#### AUDIT-C

AUDIT-C is a valid tool for detecting risky drinking and is frequently used with Indigenous Australians [13, 17, 20–22]. AUDIT-C is comprised of the first three questions from the 10-item Alcohol Use Disorders Identification Test (AUDIT) [23]. The first item is “How often do you have a drink containing alcohol?”. Responses are on a five-point scale ranging from “Never” to “4 + times per week”. The second item is “How many drinks containing alcohol do you have on a typical day when you are drinking?”. Responses to the second item are on a five-point scale ranging from “1–2” to “10 or more”. The final item is “How often do you have six or more drinks on one occasion?”. Responses are on a five-point scale and range from “Never” to “Daily or almost daily”. Each five-point scale is converted to a score from 0 to 4 (Table 1). In accordance with Australian national standards for Aboriginal health services, clients were classified as at-risk from alcohol consumption if their total AUDIT-C score was 3 or more for females, or four or more for males [13].

**Table 1:** AUDIT-C scoring table

	Score				
Questions	0	1	2	3	4
How often do you have a drink containing alcohol?	Never	Monthly or less	2–4 times per month	2–3 times per week	4 + times per week
How many standard drinks of alcohol do you drink on a typical day when you are drinking?	1–2	3–4	5–6	7–9	10 +
How often do you have 5 or more drinks on one occasion?	Never	Less than monthly	monthly	Weekly	Daily or almost daily

Item scores are summed. Women who score 3 + and men who score 4 + are at risk

### Reporting of risk assessments

Clinicians recorded their risk assessments directly into Communicare. Health professionals could use a variety of templates to input data. Sometimes AUDIT-C or an unstructured assessment could be performed on their own. On other occasions, templates would direct clinicians to perform both assessments, such as in the commonly used ‘Adult Health Check’ or ‘Pre-consult Examination’ templates. The content of these forms varies over time due to modifications made by Communicare, or due to edits performed by individual services. For example, at the start of data collection, the Adult Health Check by default only included the unstructured assessment. But at a later stage the standard Adult Health Check form asked clinicians to first make an unstructured assessment, and then to ask AUDIT-C questions. The extracted data did not tell us the ordering in which the two assessments were collected. Communicare automatically calculates AUDIT-C risk for clients and displays the result to clinicians. There is nothing preventing clinicians from changing their unstructured risk assessments based on feedback later gained from AUDIT-C.

### Data analysis

We used R version 4.1.2 (2021-11-01) [24] for our analyses. To ensure accurate transcription of results, we prepared this manuscript using the library ‘papaja’ [25]. We aggregated the dataset such that each row summarises screening activity across a two-monthly data-extraction period for a given client. We then extracted all sessions where unstructured risk, and AUDIT-C assessments were made within the same data-extraction period. Where multiple risk assessments (of the same kind) were made for a client during a single data-extraction period, the client was judged to be at risk by that assessment if half or more returned positive results. We retained the average AUDIT-C score per client over each reference period for use as a continuous variable in regressions.

Sensitivity and specificity for unstructured drinking risk assessments using AUDIT-C screening as the reference test were estimated using logistic regression [26]. As data were clustered by clients and services, we used mixed-effects models to estimate sensitivity and specificity which included random intercepts for clients and for services using the R package ‘lme4’ [27]. We derived 95% confidence intervals using the delta method function from the ‘car’ package [28]. We visualised the sensitivity and specificity of unstructured drinking risk assessments against all possible AUDIT-C thresholds using the libraries ‘ggplot2’ [29] and ‘plotROC’ [30].

To explore whether demographic factors were linked to unstructured risk assessments, we fit a series of mixed-effects logistic regressions. These multi-level models include fixed effects which estimate the effect of observed variables as well as random effects which model the effects of the unobserved characteristics of cluster variables. As observations were nested within clients who were nested within services, we included random intercepts for each client and for each service. We estimated 95% confidence intervals using the Wald approximation. In the first model we test whether AUDIT-C score significantly ($\alpha$ < 0.05) predicts clients being found at risk during unstructured risk assessments. In the second model we introduce the proportion of assessments made during the same visit as a covariate. We examined this covariate because if unstructured risk assessments are more sensitive when both assessments were performed on a single occasion, then clinicians might be adjusting their assessments based on AUDIT-C score which would confound our findings. In model 3 we additionally test for client age, gender and remoteness. In a fourth model we add an interaction between AUDIT-C score and client age. We tested for this interaction as we found a large main effect of age and because the kinds of drinking risk exhibited (single occasion vs lifetime risk) varies by age among Indigenous Australians [1]. We test whether each model significantly improved upon the previous model’s fit with Likelihood ratio tests [27]. Intraclass-correlation coefficients (ICC) for each model were estimated with the ‘performance’ library [31]. ICCs describe the percentage of variance attributable to the random effects (i.e., to heterogeneity among clients and services) [31].

## Results

### Observations

During the reference period, we observed 231,154 clinical sessions. During this period, AUDIT-C was performed on approximately one in nine clinical occasions (10.85%). Unstructured risk assessments were more frequently conducted: in approximately one in six sessions (17.11%). Across all clinical sessions, clients were more likely to be found to be at risk on occasions when their drinking was assessed using AUDIT-C (39.11%), than when unstructured risk assessments were used (8.77%). We summarised screening activity over two-monthly data extraction periods and analysed cases where both AUDIT-C and an unstructured risk assessment were conducted during the same two-monthly period.

We observed 9,884 (7.99%) instances where both AUDIT-C and an unstructured risk assessment were recorded in the same two-monthly extraction period. These data were provided by 18 services (the other four services did not record both an unstructured risk assessment and an AUDIT-C result for any client in a single extraction period). There were 6,380 unique client IDs. The mean number of observations per client ID was 1.55 ($SD$ = 1.36). Demographics for this sample are presented in Table 2. On about half of occasions (52.7%) clinicians performed unstructured risk assessments and the AUDIT-C on the same occasion. On these occasions, both items were usually featured on the same health check template in their practice management software (84.3%).

**Table 2:** Service and client characteristics for occasions where AUDIT-C score and an unstructured risk rating were recorded

Variable	Value
Service characteristics	
*n*	18
Remoteness	
Urban and inner regional	9
Outer regional and remote	4
Very remote	5
Client characteristics	
* n*^a^	6380
Observations per client (SD)	1.5 (1.4)
Age in years (SD)	38.3 (16.1)
Current drinkers	63.1%
Mean AUDIT-C score (SD)	3.1 (3.3)

*SD* standard deviation

^a^Client sample size estimated from number of unique client IDs. As clients could attended more than one service the true number of unique individuals may be lower (for most services this is unlikely; the average distance between services was 1510 km). Drinking Status established using AUDIT-C score

### Comparing AUDIT-C and unstructured drinking risk assessments

**Fig. 1:**
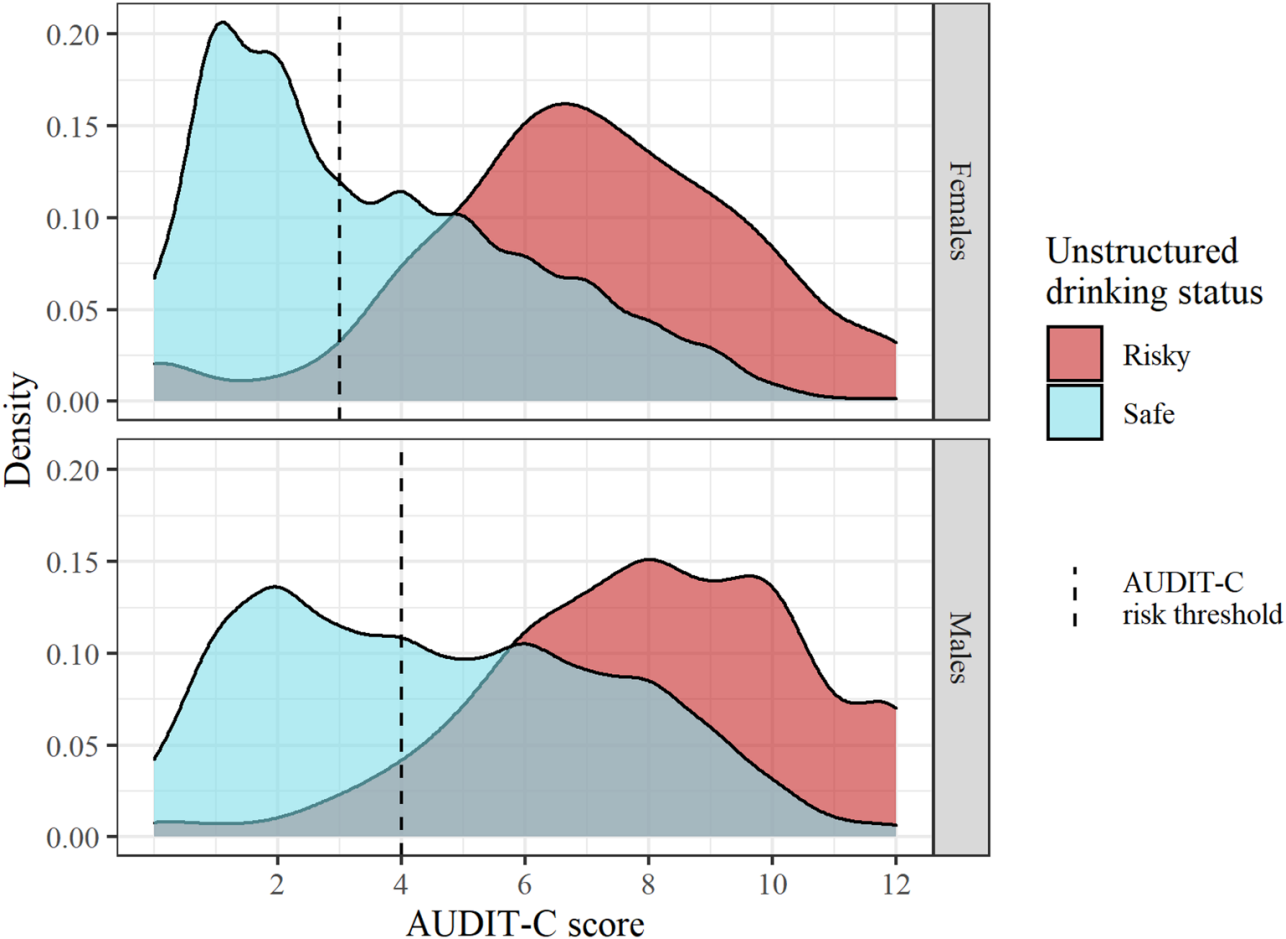
Density plot of AUDIT-C score by unstructured drinking risk rating. Sessions where clients were rated as non-drinkers by clinicians in unstructured assessments and by AUDIT-C were excluded. The area to the right of the dashed line are sessions which would be rated as risky based on AUDIT-C thresholds. Most current drinkers rated as drinking within safe limits by clinicians using unstructured assessments were classified as at risk by AUDIT-C

Unstructured risk assessments were at-odds with AUDIT-C results in more than one quarter of cases (28.74%). This level of disagreement is substantial and statistically significant ${\chi }^{2}$(1) = 2623.34; $p$ < 0.001; McNemar’s test [32]. These disagreements resulted in a greater than three-fold difference in the identified rate of risky drinking. Based on their AUDIT-C results, clients should have been classed at risk in 39.29% of periods. But using unstructured assessments, clinicians only identified clients as at risk in 11.66% of periods. Unstructured assessments showed poor sensitivity relative to classifications made with AUDIT-C (33.59% [95% CI 22.03, 47.52]), but high specificity (99.35% [95% CI 98.74, 99.67]). This means that clinicians using unstructured assessments failed to identify most people drinking at risky levels (based on AUDIT-C results). Clients who were rated as drinking ‘within safe drinking limits’ from unstructured assessments recorded a wide range of AUDIT-C scores, many of which are indicative of high-risk drinking (Fig. 1). More than half of males (60.61%) and females (55.59%) who were rated as drinking within safe limits by clinicians using unstructured assessments should have been classed as at risk based on their AUDIT-C scores.

**Fig. 2:**
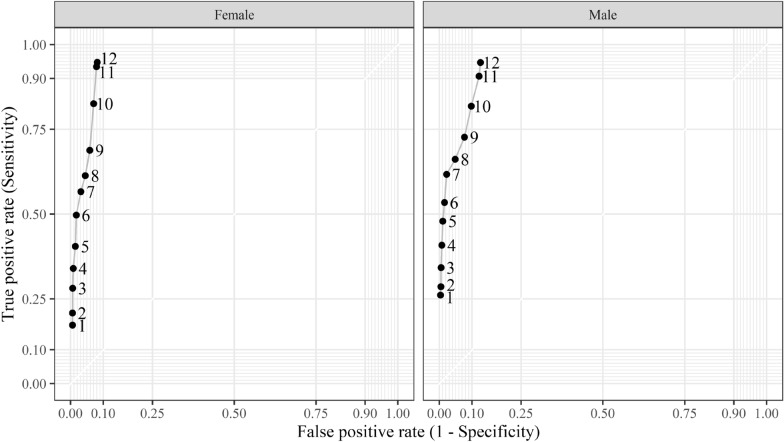
Sensitivity and specificity were estimated for unstructured risk assessments using AUDIT-C as the reference test by gender. Varying cut-offs were used to determine AUDIT-C risk (visible along each curve). These AUDIT-C cut-offs are visible as numbers along the curve. To account for clustering, sensitivity and specificity were derived from a series of multi-level logistic regressions with random intercepts for clients and services

The lack of sensitivity in unstructured assessments could mean that clinicians fundamentally disagree with AUDIT-C risk assessments. Perhaps clinicians are targeting higher cut-offs for at risk drinking. To explore this, we examined whether higher risk cut-offs can bring unstructured clinical assessments into better agreement with AUDIT-C. We estimated the sensitivity and false positive rates for unstructured risk assessments against AUDIT-C, using the full range of AUDIT-C scores as risk thresholds. Figure 2 shows that unstructured risk assessments only approached acceptable sensitivity and specificity when females and males scored 11 + on AUDIT-C. This means unstructured risk assessments only became reliable for clients at the highest levels of consumption.

### Predictors of unstructured risk ratings

To identify factors that might lead to inconsistencies between unstructured risk assessments and AUDIT-C scores, we performed a series of multi-level logistic regressions. Our data were clustered: there were multiple observations per service, as well as per client. Accordingly, we included random intercepts for both service and client ID.

**Fig. 3:**
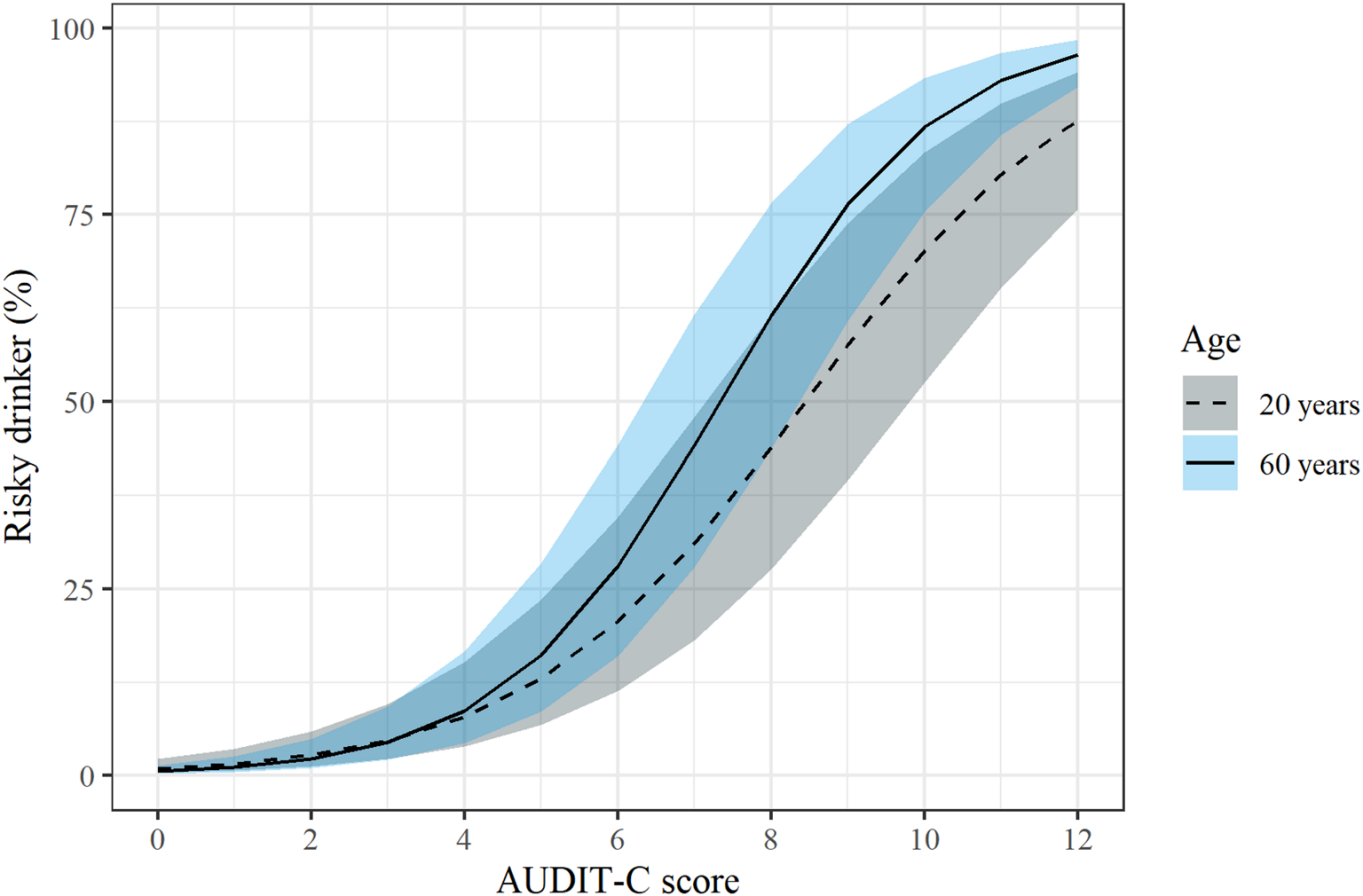
The predicted probability of being found at risk from unstructured risk assessments by AUDIT-C score and age based on Model 4. The ribbons represent 95% confidence intervals. AUDIT-C was a stronger predictor of being identified as an at-risk drinker during unstructured assessments for older clients. Model 4 controlled for remoteness, gender, and whether AUDIT-C and the unstructured risk assessment were performed in the same clinical session

The results of our regression analyses are presented in Table 3. Findings were robust to clustering by client (Additional file 1: Table S1). In the first model we identified a strong positive relationship where 1-unit increases in AUDIT-C substantially increased the odds of clients being identified as being at risk during unstructured risk assessments $\text{OR}$ = 1.87 [95% CI 1.77, 1.98]. Controlling for whether AUDIT-C and unstructured assessments were performed on the same occasion did not change the relationship between AUDIT-C and risk status (Model 2). Including client demographics improved model fit (Model 3). Specifically, a trend was detected where older clients were more likely to be identified as risky drinkers controlling for their AUDIT-C score (OR = 1.11 [95% CI 1.03, 1.19] for every 10-year increase in client age; Model 3). Including the interaction between AUDIT-C and client age further improved model fit (Model 4). The older the client, the stronger the relationship between client AUDIT-C and being identified as drinking at risk in unstructured risk assessments (Fig. 3). Across all models, substantial variation in risk assessments was attributable to clustering by service and client ($ICC$ $\approx$ 45.4%). There was not a statistically significant interaction between AUDIT-C and client gender, or AUDIT-C and service remoteness (Additional file 1: Tables S2 and S3 respectively).

**Table 3:** Multi-level logistic regression models predicting the odds of clients being found at risk in unstructured assessments

	Fixed effects					
Predictors	OR [95% CI]	lnOR	$SE$	$p$	$ICC$	Likelihood Ratio Test
**Model 1**	–	–	–	–	45.56%	–
Intercept	0.01 [0.00, 0.02]	− 4.91	0.41	< 0.001	–	
AUDIT-C	1.87 [1.77, 1.98]	0.63	0.03	< 0.001	–	
**Model 2**	–	–	–	–	45.59%	${\chi }^{2}$(1) = 0.01, $p$ = 0.90
Intercept	0.01 [0.00, 0.02]	− 4.90	0.42	< 0.001	–	
AUDIT-C	1.87 [1.77, 1.98]	0.63	0.03	< 0.001	–	
Same occasion	0.98 [0.72, 1.33]	− 0.02	0.15	0.90	–	
**Model 3**	–	–	–	–	45.56%	${\chi }^{2}$(3) = 9.14, $p$ = 0.028
Intercept	0.01 [0.00, 0.03]	− 5.06	0.85	< 0.001	–	
AUDIT-C	1.88 [1.78, 1.99]	0.63	0.03	< 0.001	–	
Age (decade)^a^	1.11 [1.03, 1.19]	0.10	0.04	0.005	–	
Remoteness	1.10 [0.47, 2.55]	0.10	0.43	0.83	–	
Male	0.87 [0.71, 1.06]	− 0.14	0.10	0.17	–	
Same occasion	1.08 [0.79, 1.47]	0.08	0.16	0.63	–	
**Model 4**	–	–	–	–	45.08%	${\chi }^{2}$(1) = 8.86, $p$ = 0.003
Intercept	0.01 [0.00, 0.03]	− 5.06	0.84	< 0.001	–	
AUDIT-C	1.87 [1.77, 1.98]	0.63	0.03	< 0.001	–	
Age (decade)^a^	0.88 [0.74, 1.04]	− 0.13	0.09	0.14	–	
Remoteness	1.11 [0.48, 2.56]	0.10	0.43	0.81	–	
Male	0.86 [0.70, 1.06]	− 0.15	0.10	0.16	–	
Same occasion	1.07 [0.79, 1.46]	0.07	0.16	0.65	–	
AUDIT-C * Age (decade)^a^	1.04 [1.01, 1.07]	0.04	0.01	0.003	–	

*OR* odds ratio, *lnOR* natural logarithm of the odds ratio (logit), *SE* standard error (of lnOR). *ICC* Intraclass-correlation coefficient—the percentage of variance attributable to the random effects

^a^Client age (a continuous variable) was divided by ten to represent decades. The age (decade) of each client was centered such that 0 represents 40 years. Likelihood ratio tests indicate whether a model significantly improves upon the fit of a simpler model. Model 2 did not significantly improve upon the fit of Model 1. Model 3 significantly improved upon the fit of Model 2. Model 4 significantly improved upon the fit of Model 3. This table presents the results of a multi-level regression. These models include both fixed (indicated by the column span) and random effects which enables clustering within the data to be modelled. The random effects include intercepts for each client (n = 4225) and service (k = 18). The thousands of random effect coefficients are not tabulated, but the percentage of variance explained by the random effects are described by the ICC statistics

## Discussion

We aimed to explore the accuracy of unstructured drinking risk assessments relative to AUDIT-C screening for Indigenous Australian clients attending Aboriginal Community Controlled Health Services (ACCHSs). While AUDIT-C is already used with Indigenous Australian clients, we demonstrated that it is much more common for clinicians to make unstructured risk assessments. This is problematic as we also demonstrated that when clinicians rely on unstructured assessments, clinicians did not identify most people who were at risk from drinking alcohol (based on their AUDIT-C score). We found that unstructured drinking risk assessments might be biased by client age: Clinicians were more responsive to elevated drinking for older clients than for younger clients. But this did not explain the large disparity between unstructured risk assessments and AUDIT-C screening results. As unstructured assessments tend to vastly underestimate drinking risk, we support the increased adoption of validated screening tools like AUDIT-C as aids for clinicians in assessing whether their Indigenous Australian clients are at risk from drinking alcohol.

We anticipated that the accuracy of unstructured drinking risk assessments might be different at services for Indigenous peoples than at mainstream services. Some Indigenous clients need clinicians to tailor their questioning around drinking to accommodate language and cultural differences [33]. But our findings demonstrated that unstructured drinking risk assessments are similarly effective across Indigenous and non-Indigenous contexts. One study of general practitioners (GPs; in mainstream settings) found that only 26.5% [16] of at-risk clients were detected using unstructured assessments. Our estimate for clinicians at ACCHSs was similar (33.59%).

It is not clear what factors influence unstructured risk assessments. In our sample, the link between drinking (AUDIT-C score) and being detected as at risk was stronger for older clients than younger clients. Perhaps the normalisation of heavy episodic drinking among younger Australians [34] contributed to some clinicians overlooking risky drinking as a phase, and not a pressing health concern. Alternatively, many diseases caused by drinking take time to develop; perhaps clinicians’ tendency to identify more older clients as at risk from drinking reflects clinical priorities which emphasise the treatment of current problems over the prevention of future ailments. Alternatively, older clients could have been more likely to have co-morbid conditions (e.g., diabetes) for which clinicians considered alcohol consumption a greater risk to patient health. These differences could also be explained by the relationships clinicians have with clients of different ages. Clinicians could have better rapport with older clients and thereby be better able to ask sensitive questions, and to discuss risk taking behaviours.

We found that the accuracy of unstructured risk assessments markedly improved for higher AUDIT-C risk cut-offs. This could indicate that clinicians at ACCHSs are using higher drinking risk thresholds in unstructured assessments than those recommended by national guidelines [13, 35]. Some general practitioners in non-Indigenous settings have expressed concerns that AUDIT-C risk thresholds are too sensitive [36]. Perhaps some clinicians are exclusively targeting Alcohol Use Disorders rather that unhealthy drinking. While the negative effects of alcohol dependence and harmful drinking can be obvious to clinicians, it is still important that hazardous drinking is addressed [37]. The links between risky drinking and health problems are well validated and important [35]. Brief interventions can help people reduce their consumption [10], thereby helping to prevent the occurrence (or exacerbation) of a range of conditions such as hypertension, diabetes, or various cancers [35].

Indigenous Australians tend to experience a heavier disease burden from chronic illnesses than others [38]. And, staff at ACCHSs provide care for more complex conditions than seen at many other services [39]. Given the competing demands faced by staff at ACCHSs [39], addressing lower-risk hazardous drinking may be challenging. Not all consultations offer good opportunities to talk with clients about alcohol consumption. Clients may become annoyed when clinicians ask about their drinking rather than address their presenting concerns [40]. Longer appointment times could help clinicians in screening for alcohol problems [41], but this could harm waiting times or require more staff. In many ACCHSs, Aboriginal Health Practitioners and nurses regularly perform preventative health assessments [42] and develop individualised care plans. Some screening using evidence-based tools could also be completed prior to consultation by the clients themselves in the waiting room using computer-based, visual, interactive surveys [33, 43, 44] which could also include brief interventions [45]. But fully automated tools might not be suitable for Indigenous Australian clients with lower computer literacy, or those who would feel more comfortable receiving less direct feedback delivered by clinicians in conversation.

Beyond resource constraints, clinicians have reported discomfort in talking to their clients about drinking (in mainstream settings) [46]. Some clinicians see addressing hazardous drinking to be the role of specialists or their clients’ families [40]. Drinking may be an especially sensitive topic for Indigenous Australians as heavy drinking has at times been used to stereotype Indigenous Australians as ‘drunks’ [33, 47]. Australian government policies have at times contributed to the stigmatisation of drinking by unilaterally restricting the sale of alcohol in various Indigenous Australian communities [47]. These sensitivities can make conversations about drinking difficult. But, for these reasons evidence-based screening tools may be especially useful in guiding conversations about drinking. Additionally, regular screening with structured assessments like AUDIT-C could normalise conversations about drinking thereby reducing stigma over time. Given the poor sensitivity of unstructured assessments, we strongly recommend the increased uptake of evidence-based screening tools such as AUDIT-C for use with Indigenous Australian clients.

Service administrators should encourage health workers to use AUDIT-C with Indigenous Australian clients instead of unstructured risk assessments where possible. Training and support for service staff can help improve uptake of AUDIT-C [17]. Training could also help health professionals in understanding what AUDIT-C results mean for their clients and how they should respond to them. In particular, based on our findings, health professionals should ensure that they do not allow younger clients with positive AUDIT-C screening results to slip through the cracks.

Finally, while AUDIT-C is a useful tool for clinicians, clinical experience and judgement are also important. Previous authors have raised questions about whether the AUDIT-C is appropriate for use with Indigenous Australian clients in remote settings [33]. Formal validation of the AUDIT-C for use with Indigenous Australians is needed. Some clients might not understand the wording of AUDIT-C items or may find answering difficult if they shared alcohol with others and did not pay attention to what they drank as an individual [33, 48]. Some Indigenous Australians might also find direct questioning based on AUDIT-C items intrusive [33]. For these situations clinicians may need to adapt AUDIT-C phrasing as needed [49], or to obtain the relevant information through conversational approaches [45]. If the basic AUDIT-C content is covered (which establishes the quantity and frequency of regular consumption, and the frequency of heavy drinking occasions), we would expect these assessments to still be effective in establishing drinking risk.

### Limitations

The accuracy of our findings relies on accurate record keeping at participating services. Since 2017 the Australian government has changed reporting requirements at ACCHSs to include the numbers of clients for which AUDIT-C was conducted [13]. Thus, the frequency of AUDIT-C assessments at ACCHSs may now be higher than what is reported here [17]. The order that clinicians performed AUDIT-C and unstructured assessments could be important in cases where clinicians performed both assessments on single occasions. However, controlling for instances where both assessments were performed during the same clinical session did not meaningfully change the relationship between AUDIT-C and finding clients at risk from drinking with unstructured assessments. This might suggest clinicians generally do not change prior unstructured assessments based on subsequent AUDIT-C results. Screening behaviour might vary between clinicians who preferred AUDIT-C to using unstructured assessments. We did not have access to clinician IDs which would be required to examine this, or to model clustering by clinician. We encourage future work which clarifies to what degree clinical preferences drive screening test performance. A trial where two clinicians separately assess the same clients and are randomised to either use AUDIT-C or subjective judgement could eliminate potential confounds and provide a stronger level of evidence. It is possible that repeated screening with AUDIT-C reduced its sensitivity (to true drinking risk) as has been demonstrated in samples of veterans in North America [50]. A longitudinal study comparing repeated AUDIT-C screening results to biomarkers associated with heavy drinking (e.g., blood pressure and liver enzymes) would be useful in clarifying whether repeated screening affects the accuracy of AUDIT-C screening among Indigenous Australians.

## Conclusion

Unstructured drinking risk assessments are commonly made at Aboriginal Community Controlled Health Services. Most people drinking at risky levels (based on AUDIT-C) are not identified when clinicians use unstructured assessments. Increasing use of AUDIT-C would help detect substantially more clients at risk from alcohol consumption. Detection of hazardous consumption provides opportunities to engage clients in conversation on the effects of alcohol consumption on their health.

## Supplementary Information

**Additional file 1: Table S1**. The odds of clients being found at risk in unstructured assessments. One observation used per client (the first). **Table S2**. The odds of clients being found at-risk using unstructured screening by age, gender, AUDIT-C score, remoteness, same occasion screening, and the interaction between AUDIT-C and gender. **Table S3**. The odds of clients being found at-risk using unstructured screening by age, gender, AUDIT-C score, remoteness, same occasion screening, and the interaction between AUDIT-C and service remoteness.

## Data Availability

Data for this project is stored at the University of Sydney based at Drug Health Service, KGV Building, Missenden Road, Camperdown New South Wales, 2050 Australia.
