# Supplementary material for: More than three times as many Indigenous Australian clients at risk from drinking could be supported if clinicians used AUDIT-C instead of unstructured assessments
Source: Addict Sci Clin Pract. 2022 Apr 5;17:23. doi: 10.1186/s13722-022-00306-5 (PMC8981780; doi:10.1186/s13722-022-00306-5)
Supplement: Supplementary file 1 — Additional file 1: Table S1. The odds of clients being found at risk in unstructured assessments. One observation used per client (the first). Table S2. The odds of clients being found at-risk using unstructured screening by age, gender, AUDIT-C score, remoteness, same occasion screening, and the interaction between AUDIT-C and gender. Table S3. The odds of clients being found at-risk using unstructured screening by age, gender, AUDIT-C score, remoteness, same occasion screening, and the interaction between AUDIT-C and service remoteness. [file 13722_2022_306_MOESM1_ESM.pdf]

Supplementary Materials: More than three times as many Indigenous Australian clients at risk from drinking could be supported if clinicians used AUDIT-C instead of unstructured assessments

James H. Conigrave[1,2], K.S. Kylie Lee[1,2,3,4,5], Paul S. Haber[1,2,6], Julia Vnuk[7,8], Michael F. Doyle[1,2], & Katherine M. Conigrave[6,1,2]

[1] NHMRC Centre of Research Excellence in Indigenous Health and Alcohol, Faculty of Medicine and Health, Central Clinical School, Discipline of Addiction Medicine, New South Wales, Australia
[2] The Edith Collins Centre (Translational Research in Alcohol, Drugs and Toxicology), Sydney Local Health District, New South Wales, Australia
[3] National Drug Research Institute, Faculty of Health Sciences, Curtin University, Western Australia, Australia
[4] La Trobe University, Centre for Alcohol Policy Research, Victoria, Australia
[5] Burnet Institute, Victoria, Australia
[6] Royal Prince Alfred Hospital, Drug Health Services, New South Wales, Australia
[7] Aboriginal Health Council of South Australia, South Australia, Australia
[8] Adelaide Rural Clinical School, The University of Adelaide, South Australia, Australia

This document presents sensitivity analyses for our logistic regression models predicting unstructured risk by client and service characteristics

Restricting client observations

We limited each client to only have one observation to see if our results were sensitive to clustering by client. Table S1 presents the results of this sensitivity analysis. The pattern of results was unchanged and demonstrates our main findings are robust to clustering by client. By limiting clients to only having one observation heterogeneity was meaningfully reduced (as seen in the ICCs).

Correspondence concerning this article should be addressed to James H. Conigrave, Level 6, King George V Building, Drug Health Services, 83-117 Missenden Road, Camperdown NSW 2050, Australia. E-mail: james.conigrave@sydney.edu.au

Table S1

The odds of clients being found at risk in unstructured assessments. One observation used per client (the first).

	Fixed effects					
Predictors	OR [95% CI]	lnOR	*SE*	*p*	*ICC*	Likelihood Ratio Test
Model 1	-	-	-	-	29.15%	-
Intercept	0.02 [0.01, 0.03]	-4.17	0.33	< 0.001	-	
AUDIT-C	1.70 [1.64, 1.78]	0.53	0.02	< 0.001	-	
Model 2	-	-	-	-	29.20%	$\chi^2(1) = 0.09$, $p = 0.76$
Intercept	0.02 [0.01, 0.03]	-4.14	0.34	< 0.001	-	
AUDIT-C	1.70 [1.64, 1.78]	0.53	0.02	< 0.001	-	
Same occasion	0.96 [0.71, 1.28]	-0.05	0.15	0.76	-	
Model 3	-	-	-	-	29.65%	$\chi^2(3) = 6.82$, $p = 0.078$
Intercept	0.01 [0.00, 0.06]	-4.23	0.73	< 0.001	-	
AUDIT-C	1.72 [1.65, 1.79]	0.54	0.02	< 0.001	-	
Age (decade)†	1.08 [1.01, 1.16]	0.08	0.04	0.032	-	
Remoteness	1.06 [0.51, 2.20]	0.06	0.37	0.88	-	
Male	0.85 [0.70, 1.03]	-0.17	0.10	0.095	-	
Same occasion	1.02 [0.76, 1.37]	0.02	0.15	0.90	-	
Model 4	-	-	-	-	29.45%	$\chi^2(1) = 8.25$, $p = 0.004$
Intercept	0.01 [0.00, 0.06]	-4.25	0.73	< 0.001	-	
AUDIT-C	1.72 [1.65, 1.80]	0.54	0.02	< 0.001	-	
Age (decade)†	0.86 [0.73, 1.02]	-0.15	0.09	0.091	-	
Remoteness	1.06 [0.51, 2.21]	0.06	0.37	0.87	-	
Male	0.84 [0.69, 1.02]	-0.17	0.10	0.084	-	
Same occasion	1.00 [0.74, 1.36]	0.00	0.15	0.98	-	
AUDIT-C * Age (decade)†	1.04 [1.01, 1.06]	0.04	0.01	0.005	-	

Note. SE = standard error (of lnOR). *ICC* = Intraclass-correlation coefficient. † Client age (a continuous variable) was divided by ten to represent decades. The age (decade) of each client was centered at 40 years.

Table S2

The odds of clients being found at-risk using unstructured screening by age, gender, AUDIT-C score, remoteness, same occasion screening, and the interaction between AUDIT-C and gender.

Predictors	lnOR	*SE*	OR [95% CI]	*p*
Intercept	-5.16	0.86	0.01 [0.00, 0.03]	< 0.001
Age (decade)†	0.10	0.04	1.11 [1.03, 1.19]	0.004
Male	0.07	0.26	1.07 [0.64, 1.79]	0.79
AUDIT-C	0.65	0.04	1.92 [1.78, 2.06]	< 0.001
Remoteness	0.09	0.43	1.10 [0.47, 2.55]	0.83
Same occasion	0.07	0.16	1.08 [0.79, 1.47]	0.64
AUDIT-C * Male	-0.03	0.04	0.97 [0.90, 1.04]	0.37
Random Effects				
τ_{00} id:service	0.92			
τ_{00} service	1.84			
ICC	45.60%			

Note. SE = standard error (of lnOR). † Client age was centered such that 0 represents 40 years.

Interaction between AUDIT-C and gender

We present an additional model examining whether client gender interacts with AUDIT-C to predict unstructured risk assessments. This additional coefficient was not a significant predictor of unstructured risk assessment (Table S2).

Interaction between AUDIT-C and remoteness

We present an additional model examining whether service remoteness interacts with AUDIT-C to predict unstructured risk assessments. This additional coefficient was not a significant predictor of unstructured risk assessment (Table S3).

Table S3

The odds of clients being found at-risk using unstructured screening by age, gender, AUDIT-C score, remoteness, same occasion screening, and the interaction between AUDIT-C and service remoteness.

Predictors	lnOR	*SE*	OR [95% CI]	*p*
Intercept	-5.34	0.88	0.00 [0.00, 0.03]	< 0.001
Age (decade)†	0.10	0.04	1.11 [1.03, 1.19]	0.005
Male	-0.15	0.10	0.86 [0.70, 1.06]	0.15
AUDIT-C	0.68	0.05	1.97 [1.78, 2.18]	< 0.001
Remoteness	0.27	0.45	1.31 [0.54, 3.19]	0.55
Same occasion	0.08	0.16	1.08 [0.79, 1.47]	0.62
AUDIT-C * Remoteness	-0.03	0.03	0.97 [0.92, 1.02]	0.25
Random Effects				
τ_{00} id:service	0.89			
τ_{00} service	1.82			
ICC	45.20%			

Note. SE = standard error (of lnOR). † Client age was centered such that 0 represents 40 years.
